# *QuickStats*: Percentage* of U.S. Women Aged 50–74 Years Who Never Had a Mammogram,^†^ by Place of Birth and Length of Residence in the United States^§^ — National Health Interview Survey, 2013 and 2015^¶^

**DOI:** 10.15585/mmwr.mm6611a8

**Published:** 2017-03-24

**Figure:**
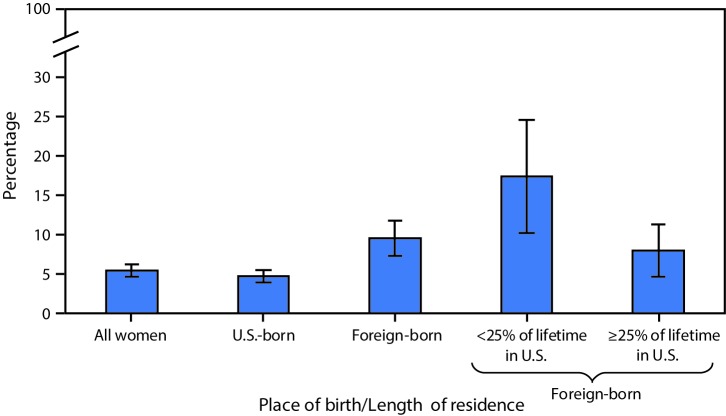
In 2013 and 2015 combined, 5.4% of U.S. women aged 50–74 years had never received a mammogram in their lifetime. Foreign-born women were twice as likely as U.S.-born women to have never received a mammogram (9.5% versus 4.7%). Foreign-born women who lived in the United States for <25% of their lifetime were more than twice as likely to have never received a mammogram compared with those who resided in the U.S. for ≥25% of their lifetime (17.3% versus 7.9%).

**Source:** National Health Interview Survey, 2013 and 2015 combined. https://www.cdc.gov/nchs/nhis.htm.

